# Chimeric antigen receptor T‐cell therapy: Challenges and framework of outpatient administration

**DOI:** 10.1002/jha2.333

**Published:** 2021-11-19

**Authors:** Katie S. Gatwood, Bhagirathbhai R. Dholaria, Mariana Lucena, Brittney Baer, Bipin N. Savani, Olalekan O. Oluwole

**Affiliations:** ^1^ Department of Pharmacy Vanderbilt University Medical Center Nashville Tennessee USA; ^2^ Division of Hematology‐Oncology Department of Medicine Vanderbilt University Medical Center Nashville Tennessee USA; ^3^ Medexus Pharmaceuticals, Inc. Chicago Illinois USA; ^4^ Department of Nursing Clinical Trials Office Vanderbilt University Medical Center Nashville Tennessee USA; ^5^ Division of Hematology‐Oncology Department of Medicine Tennessee Valley Healthcare System Nashville Tennessee USA

**Keywords:** chimeric antigen receptor T cell, leukemia, lymphoma, monitoring, outpatient, toxicity

## Abstract

Adoptive cellular therapy has made a landmark change within the treatment paradigm of several hematologic malignancies, and novel cellular therapy products, such as chimeric antigen receptor T‐cell therapy (CART), have demonstrated impressive efficacy and produced durable responses. However, the CART treatment process is associated with significant toxicities, healthcare resource utilization, and financial burden. Most of these therapies have been administered in the inpatient setting due to their toxicity profile. Improved toxicity management strategies and a better understanding of cellular therapy processes are now established. Therefore, efforts to transition CART to the outpatient setting are warranted with the potential to translate into enhanced patient quality of life and cost savings. A successful launch of outpatient CART requires several components including a multidisciplinary cellular therapy team and an outpatient center with appropriate clinical space and personnel. Telemedicine should be incorporated for closer monitoring. Additionally, clear criteria for admission upon clinical decompensation, a pathway for prompt inpatient transition, and clear toxicity management guidelines should be implemented. Effective education about cellular therapy and toxicity management is imperative, especially for the Emergency Department and Intensive Care Unit teams. Here, we have outlined the various logistical and clinical considerations required for the care of CART patients, which will aid centers to establish an outpatient CART program.

## 1 INTRODUCTION

Chimeric antigen receptor therapy (CART) has shown efficacy in the treatment of high‐grade B cell lymphoma, acute lymphoblastic leukemia, mantle cell lymphoma, follicular lymphoma, and multiple myeloma [1, 2, 3]. This has led to several CART products being approved by the U.S. Food and Drug Administration (FDA). CART therapies are rapidly changing the paradigm of treatment for wide‐ranging hematological malignancies. Multiple challenges need to be overcome for this nascent treatment process to be expanded to the vast areas of unmet medical need. Although the earliest registration trials were done in the inpatient setting due to concerns around cytokine release syndrome (CRS) and immune effector cell‐associated neurotoxicity syndrome (ICANS), the toxicity management process has improved greatly over the years and the rates of acute higher grade toxicity have dropped significantly with early intervention with corticosteroids and tocilizumab [4, 5]. The ongoing evolution of management guidelines and its rapid adoption to clinical practice calls into question whether inpatient therapy with its attendant cost is truly necessary for all CART recipients [4]. It is anticipated that CART therapy will eventually be an outpatient process at least for the products where we have clear evidence‐based management guidelines for early intervention. Optimal patient selection is also a key in excluding the patients who are at high risk for complications after CART therapy in the outpatient setting.

Outpatient therapy can be provided in the ambulatory clinic setting where equipment and personnel are available to deal with semiemergent situations like infusion reactions. It is also open for an extended period but not continuously for 24 h. All the currently approved CART products are autologous with little risk of immediate infusion reaction, although premedication is always given. This suggests that the infusion of the product can be done in the clinic setting.

A successful outpatient program will have mastery of the time frame that each potential complication of CART is likely to occur, and institute‐specific measures for early detection and intervention. It will also have suitable lodging facilities that are sufficiently proximal to the hospital, reliable caregiver support, a direct line of communication to the clinical team, and immediate availability of inpatient beds to admit based on specific symptoms and signs. It will also have a seamless system for rapid resuscitation and administration of tocilizumab, corticosteroids, and extended‐spectrum antibiotics.

## 2 HISTORICAL PERSPECTIVE AND RATIONALE

Cellular therapy with CART falls under the auspices of the newly created immune effector cell therapy category. Autologous and allogeneic hematopoietic stem cell transplant (HCT) is a form of cellular therapy that has become standard of care for many diseases for many years and many centers are routinely performing HCT in the outpatient setting.

Some data suggest that outpatient HCT can be done as safely as in the inpatient setting [6, 7]. Outpatient HCT can be more cost efficient and may be associated with improved patient satisfaction compared to inpatient HCT [6, 7].

There are a few published reports where CART was said to have been given in the outpatient setting. Abramson reported that 25 of 344 patients received their CART in the outpatient setting. The low number suggests that these were highly selected patients and by far the minority of those who could have been treated [8].

## 3 COMPONENTS OF AN OUTPATIENT PROGRAM

### 3.1 Regulatory

Traditionally, HCT is regulated by the Foundation for the Accreditation of Cellular Therapy (FACT) standards. CART therapy in many ways follows a similar workflow as traditional HCT such as donor workup, apheresis collection, labeling, storage, documentation, and product administration. Currently, FACT is encouraging certification for immune effector cell (IEC) therapy if a program is administering research or commercial CART products. However, getting FACT IEC accreditation is not mandated for Centers for Medicare & Medicaid Services if a program is meeting the requirements for Risk Evaluation and Mitigation Strategy (REMS) program implemented by each CART product (National Coverage Analysis, decision memo CAG‐00451N). An increasing number of industry‐sponsored CART studies are requiring FACT accreditation, but the practice has been variable. Meeting the REMS requirements is more feasible for independent community‐based practices without affiliation with the FACT accredited HCT program. In the future, we hope to see further consolidation of REMS requirements to reduce the regulatory burden. Implementation of outpatient CART program will require meeting these FACT IEC standards and/or REMS requirements, which includes appropriate training of clinical staff, maintaining training logs, reporting adverse events from CART therapy, verifying availability of tocilizumab, and complying with audits.

The goal of developing an outpatient CART program is to deliver optimal patient care and at the same time utilize healthcare resources judiciously. There are many reasons why outpatient therapy is preferred if it can be done as safely as in the inpatient setting [9, 10]. Many hospitals operate under conditions in which they must be judicious with the use of resources. This lack of redundancy makes it problematic to accommodate unplanned admissions which are likely to occur in patients receiving CART in the outpatient setting. On a positive note, the ability to treat patients in the outpatient setting will free up hospital beds that can then be used to care for other patients with acute complications.

There are also economic factors that need to be considered. For example, Medicare reimbursement for CART therapy done in the inpatient setting often leaves the healthcare facility at a cost deficit. Contrarily, outpatient care does not attract nearly as much of a deficit. Reimbursement contracts are less favorable if patients end up being admitted within 72 h of receiving a drug (including CART) because the institution is reimbursed at a lower rate. Additionally, each center may have different workflows in terms of acquiring insurance approval for tocilizumab. Some centers can bundle the authorization as part of the entire CART administration case agreement or case rate. Other centers may need to submit a claim for tocilizumab coverage separately and ensure it is approved in anticipation of needing to use it emergently in the outpatient setting.

### 3.2 Personnel

A cohesive cellular therapy multidisciplinary healthcare team is by far the most important component of an outpatient cellular therapy program. This core group should consist of multiple entities including cellular therapy physicians plus a medical director, cellular therapy coordinators, advanced practice providers (APPs), clinical and research nurses, financial coordinators, apheresis and cellular therapy processing lab personnel, clinical pharmacy specialists, social workers, case managers, and procurement personnel. Alliances to this central group include pharmacy informatics, emergency department (ED) staff and intensive care unit (ICU) staff, and hospitalists [11].

The members of the core CART team will assume certain roles and responsibilities, but the cellular therapy program should ensure each responsibility has a designated member to be carried out appropriately. The CART physicians and APPs should confirm that patients meet the criteria to proceed with outpatient treatment. These providers will be involved in monitoring and managing CART‐related toxicities. Once the need for cellular therapy is identified, the financial and procurement coordinators should be in charge of developing case rates/agreements, placing orders for the products, and verifying the submission and reimbursement of claims. The case managers and social workers are primarily focused on arranging plans for dedicated caregivers and local housing if patients need temporary housing near the CAR T center. The CART coordinators are involved in scheduling patient visits, educating patients and caregivers on appropriate on‐call contacts, and logistics regarding the treatment. The apheresis and cellular therapy processing lab personnel will work together to collect, process, ship, store, and transfer the CART products to the outpatient center. They are also fundamental in coordinating the time of cell infusion given most cryopreserved CART products have a short expiration time after thawing. The clinical pharmacy specialists are involved in creating and verifying the lymphodepleting chemotherapy orders. They also serve as key educators to the patients, caregivers, and cellular therapy team on adverse event management and supportive care strategies. The pharmacists are also involved in securing enough tocilizumab supply for patients and communicating with procurement personnel to maintain appropriate inventory. In some centers, the pharmacists may also be tasked with ensuring the cellular therapy program meets REMS program requirements [12, 13, 14, 15, 16]. Depending on state regulations, pharmacists may also be required to label the cellular product including the CART product before it is infused into the patient. Nurses are tasked with the administration of CART products and supportive care medication and coordination of the time of infusion with the cellular therapy lab personnel. The cellular therapy program should also have a contingency plan implemented for treating patients who remain ineligible for outpatient CART for any reason.

Each member of the CART treatment team is an invaluable asset in supporting outpatient treatment. However, alliances should be forged with other healthcare groups whose expertise comes is leveraged when patients require immediate intervention for toxicities such as CRS and/or ICANS. If patients or caregivers recognize worsening symptoms of CRS and/or ICANS, they could present to the ED, prompt a direct admission to the ICU, or be admitted to the inpatient oncology/CART unit. Healthcare professionals staffing ED and ICU should be aware of the severity of CART therapy‐associated toxicities as they can oftentimes present similar to sepsis or encephalopathy; they need to recognize the need for tocilizumab or corticosteroids and have quick access to CART specialists on‐call [17, 18]. Therefore, the CART team should engage with key leaders of the ED, ICU, and hospitalists to provide education specific to CART and anticipated toxicities. Effective communication among these teams is crucial for appropriate medical management. The ED, ICU, and hospitalist teams should be provided with a point of contact designated to discuss the need for tocilizumab or corticosteroids. The way this is done may vary from center to center. One example is to create a virtual pager that is forwarded to the on‐call CART provider when he/she is covering the inpatient service. This can ensure a single pager number is distributed to the ED, ICU, and hospitalist teams instead of multiple pagers or staff numbers that can lead to confusion. Furthermore, depending on the institution's electronic medical record (EMR) certain features could be attached to the patient's profile serving as alerts to any provider. For example, the patient's header on the EMR chart may be flagged with a different color or an additional text box stating “CAR T‐cell therapy patient.” Another feature may be to create a "complex condition letter" with information on the type of CART therapy/product, date of infusion, anticipated toxicities and management, and cell therapy on‐call provider pager. This letter can then be added to a best practice alert that can inform providers, remind them of prior education, and provide appropriate CART resources. Similarly, an alert to prevent the administration of corticosteroids for reasons other than ICANS could be added to the patient's chart by including corticosteroids as a conditional allergy for a specified timeframe. If tocilizumab or corticosteroids are warranted, they must also be promptly ordered and dispensed for administration. Corticosteroids are easily available in most hospital departments, but the clinical pharmacy specialist and nursing teams can ensure that the most readily used agents (e.g., intravenous dexamethasone and methylprednisolone) are available in the Pyxis or Omnicell stations. Ordering of corticosteroids is typically not restricted, but the correct doses and frequencies for CRS and ICANS should be reinforced through educational materials. The acquisition of tocilizumab faces more challenges but can be appropriately ordered with education and informatics support. Each institution may have distinct oncology‐based EMR systems (e.g., EPIC Beacon) allowing for different functionalities for chemotherapy orders. Per REMS program requirements, tocilizumab should be administered immediately (within 2 h of observed toxicity) [17, 19]. Pharmacy informatics specialists could help build tocilizumab orders to be available for quicker dispensing and administration. For example, individual tocilizumab orders with the correct dose, indication, and frequency could be placed within EPIC Beacon treatment plan orders to appear daily for hematology/oncology providers to access. For non‐EPIC Beacon providers, an order panel with the correctly prepared tocilizumab order could be created for easier searching and access. The coordination of the dispensing, compounding, and administration of tocilizumab between providers, pharmacists, and nurses is crucial for prompt delivery and infusion. Education on REMS, tocilizumab ordering, and workflow is paramount for appropriate CART patient management.

### 3.3 Infrastructure

The implementation of a successful outpatient CART program requires significant logistical planning and execution. Regarding the physical location, an outpatient cellular therapy center should be a dedicated place for the primary components of cellular therapy including lymphodepletion therapy, infusion of cellular products, patient and provider visits, and toxicity monitoring and management. Ideally, the center should have open hours seven days a week for at least daily patient visits for the first two weeks postinfusion to ensure specialized providers can evaluate and manage potential toxicities, for example, CRS and ICANS in patients receiving CART products [17, 19]. If the dedicated infusion center does not support weekend open hours, an alternative option is for the team to offer telemedicine visits provided by on‐call physicians, hematology/oncology fellows, or APPs.

The outpatient CART program should be able to support clinic visits to the outpatient unit daily in the morning with the purpose to do a complete physical exam including a detailed neurological exam, review of systems, and laboratory tests. If patients are asymptomatic, they can safely be sent back to their local housing. Institutions may have their individualized set of parameters that guide admission to the hospital [20]. Since the period of neutropenia generally coincides with the development of CRS, such patients should be considered for inpatient evaluation for fever (≥38°C) and sepsis rule‐out. However, selected patients who have been evaluated may also be managed as an outpatient with close monitoring if all other vital signs are stable and low concern for systemic infection. Potential strategies include starting tocilizumab combined with a short course of dexamethasone 10 mg/day for 3 days for grade 1 CRS or dexamethasone 10 mg/day for 3 days for grade 1 ICANS in the appropriate instance per institution practice [18].

The process for admission will also vary by institution. One recommendation is to implement a "scatter" hospital bed that can be made available for the patient to be admitted. Additionally, this bed should be in a designated unit where specific interventions like tocilizumab and high‐dose corticosteroids are available. Admission through the ED is an option so long as the ED is set up as able to rapidly evaluate the patient and administer specific interventions. The ED physicians will also need to be REMS trained and keep up with other regulatory requirements.

Telemedicine visits are encouraged for patients undergoing outpatient CART to help monitor toxicities during the after hours. Depending on the institution's bandwidth and staffing, telemedicine visits can be coordinated once or twice a day as well as on weekends if the center is not open 7 days per week. Telemedicine should include both audio and video components to do a review of systems and focused physical exam. A basic neurological functional assessment can be done including viewing a signature. These telemedicine visits can help to identify signs and symptoms of toxicities including CRS/ICANS and guide the patient to appropriate management strategies (e.g., continue to monitor, or direct to hospital for administration of tocilizumab and/or corticosteroids).

Additionally, some centers may be able to offer patients wearable devices. They can be worn for prolonged periods (for up to 30 days), and they can give valuable real‐time data that are actionable. It is recommended to use devices that measure at least heart rate, body surface temperature, and blood pressure. Furthermore, some CAR T‐cell therapy pharmaceutical companies provide support for outpatient treatments with digital platforms to provide pertinent information on personalized products and patient support (e.g., Cell Therapy 360) [21].

**FIGURE 1:**
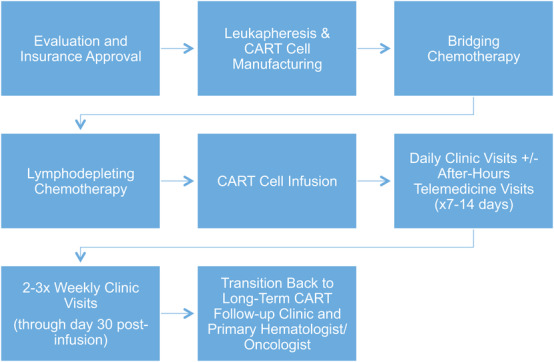
Outpatient cellular therapy workflow for chimeric antigen receptor T‐cell therapy (CART)

As with a variety of other clinical applications, SOPs and policies should be created to document and follow appropriate practices. Figure 1 outlines the basic schema of patient journey during outpatient CART therapy. It is recommended that the cell therapy program builds the SOPs outlining the studies and evidence for CART therapy, patient selection, criteria and eligibility for outpatient administration, assigned staffing roles and responsibilities, patient follow‐up requirements, toxicity management, and supportive care strategies. These policies will help the CART program ensure the internal guidelines are followed for each patient undergoing outpatient CART. Some institutions have adopted the use of quick reference guides to have available for certain diseases, indications, therapeutic management and one can be created for outpatient cellular therapy workflow. Nevertheless, cellular therapy is changing rapidly and new findings from clinical experience may lead to changes for improved patient management. Thus, the SOPs and policies are dynamic and should be subject to annual revisions to fine‐tune any outdated areas.

## 4 SETTING UP THE STRUCTURE

Education is imperative to having a successful CART program. Education should take place in all areas of the treatment facility where CAR T patients may be treated, including the ED. Any staff that will be caring for these patients will be required to be REMS certified as stated in previous sections. Individual roles of providers should be clearly defined. It is beneficial to have a specific coordinator that will be responsible for these CART patients that will help patients, caregivers, and the facility navigate the logistics of CART therapy. The CART coordinator is responsible for coordinating with the pharmaceutical company to secure manufacturing dates for the cellular products and communicating these dates with the treating facility's ancillary departments to ensure adequate time to schedule. A designated service line for overseeing CART patients can be helpful by providing the extra oversight in taking care of these patients and their unique adverse events. While the CART service line may not be the primary admitting team for these CART patients, they should be a consulting service to oversee the CART‐related side effects. This service line should be composed of both physicians and APPs.

Historically, CART administration has fallen under the purview of HCT, but as the number of FDA‐approved CART products increases in the future, there will be an increased demand to build programs that are specifically focused on CART and other immune effector cell products. If the CART patients are going to be ultimately treated in the HCT service, all the providers should be trained in CART therapy and its associated side effects.

Patient selection is important to ensure safety and minimize the risk of complications. Programs should establish criteria that patients must meet to be eligible for outpatient CART therapy. Accreditation requirements for CART programs require periodic team meetings to review potential patients for eligibility [20]. It is also imperative to conduct periodic evaluations to ensure that they remain eligible as disease status may change quickly with some leukemias and high‐grade lymphomas. Carefully selected bridging therapy may help reduce tumor growth in certain instances [20, 22].

Currently, the Center for International Blood & Marrow Transplant Research (CIBMTR) collects data that pertains to quality assurance for not only stem cell transplant patients, but also CART patients. This data is typically reported by each facility's quality manager/quality team. In the process of building a CART program, there should be a plan for having a well‐equipped quality team. The team will also need to establish which data points, in addition to the CIBMTR data points, that the facility also wants to capture, if any.

## 5 MONITORING, EXPANDING, AND METRICS FOR SUCCESS

Evaluating metrics with outpatient CAR T‐cell therapy programs will provide meaningful points of data for performance as well as areas of expansion or improvement. Immune effector cell accreditation is recommended to be able to provide cellular therapies at cancer centers, and thus periodic reviews should be performed to be able to meet the regulatory standards. Beyond these requirements, cellular therapy programs could have internal outcomes to measure such as rates and grades of CRS/ICANS, number of doses of tocilizumab and corticosteroids, time to tocilizumab administration, rate of hospital admission, and rates and types of infections. Cancer centers can adopt additional metrics to track as they gain experience with outpatient CAR T‐cell therapy for improved quality [11, 20, 23, 24].

Furthermore, the data metrics are essential to leverage additional resources for the cellular therapy program. Increased patient volume in the outpatient services may require a redistribution of resources or increased healthcare professional positions to support the growing cellular therapy program. For example, a business plan to request additional pharmacists, nurses, APPs, or physicians for an area of unmet need may include these program metrics. These may be especially more indispensable with added telemedicine responsibilities among cellular therapy program personnel. Additionally, tocilizumab inventory may need to be redistributed to have adequate supply per REMS requirements for both outpatient and inpatient CART patients [11, 20].

## 6 CONCLUSION

Outpatient administration and monitoring represent the future for CART. Being able to administer and monitor CART in the outpatient setting requires careful planning and attention to detail to preserve patient safety. It also requires appropriate oversight and adaptability to optimize patient satisfaction. The method of dealing with emergencies should be well thought out and systems in place to rapidly intervene, including admission to the hospital at the first sign of toxicity whether day or night. Quality metrics need to be developed and tracked (e.g., time from fever to tocilizumab). Hospital resource utilization will also be reduced, and the financial implications of cellular therapy delivery will show a more favorable profile. It is anticipated that the distance to the hospital will be extended, and more patients will be able to access this life‐saving intervention from the comfort of their homes.

The implementation of outpatient CART requires an outpatient infusion center able to support the needs of the patients during each phase of treatment. Most importantly, the cellular therapy program should have a core workgroup of members with dedicated roles and responsibilities to provide optimal care for these patients from start to finish. The members encompassing this workgroup consist of cellular therapy physicians, cellular therapy coordinators, APPs, nurses, financial coordinators, apheresis and cellular therapy lab personnel, clinical pharmacy specialists, social workers, case managers, and procurement personnel. Pharmacy informatics, emergency department staff, and ICU staff, and hospitalists are also healthcare professionals who may care for these patients and will require education on how to manage toxicities of cellular therapies including CART. Additional steps may be necessary to logistically prepare and administer tocilizumab and other supportive care agents for non‐oncology providers. Institutions should also incorporate telemedicine visits to be able to guide outpatient CART patients if signs/symptoms of CRS or ICANS arise. Wearable technology and other pharmaceutical company‐specific platforms could be useful to track vital signs on these patients. The cellular therapy program should also document the workflow for outpatient cellular therapy via policies and SOPs and perform routine updates with dynamic changes based on experience. Additionally, successful implementation of an outpatient cellular therapy program requires extensive patient and caregiver education as the share of responsibility in providing care shifts substantially onto the patient and caregiver. All members of the healthcare team should be involved in providing this education at multiple crucial time points along the cellular therapy journey.

## CONFLICT OF INTEREST

BNS and BB report no COI.

ML is employed by Medexus Pharmaceuticals.

KSG reports speaker bureau with Jazz pharmaceuticals and grant funding from Astra Zeneca.

OOO reports Consultancy with Pfizer, Novartis, Janssen, Kite, Gilead, Spectrum, Bayer and Curio science. Research funding with Kite.

BRD reports institution research funding from Takeda, Janssen, Angiocrine, Pfizer, Poseida, MEI, Sorrento. Consultancy from Jazz, Celgene and Gamida Cell.
